# Integration of non-invasive and conventional systems for quality assessment and authentication of meat stuffs: A review with bibliometric analysis

**DOI:** 10.1016/j.fochx.2025.102778

**Published:** 2025-07-10

**Authors:** Asima Saleem, Aysha Imtiaz, Sanabil Yaqoob, Muhammad Awais, Kanza Aziz Awan, Hiba Naveed, Ibrahim Khalifa, Sezai Ercisli, Robert Mugabi, Saqer S. Alotaibi, Gulzar Ahmad Nayik, Jian-Ya Qian, Qing Shen

**Affiliations:** a School of Food Science and Engineering, Yangzhou University, Yangzhou, Jiangsu 225127, China; b Panvascular Diseases Research Center, The Quzhou Affiliaed Hospital of Wenzhou Medical University, Quzhou People's Hospital, Quzhou 324000,China; c Laboratory of Food Nutrition and Clinical research, Institute of Seafood, Zhejiang Gongshang University, Hangzhou 310012, China; d Department of Food and Nutritional Sciences, Faculty of Science and Technology, University of Central Punjab, Lahore, Pakistan; e National Institute of Food Science and Technology, Faculty of Food, Nutrition and Home Sciences, University of Agriculture Faisalabad, Pakistan; f Food Technology Department, Faculty of Agriculture, Benha University, 13736 Moshtohor, Qaluobia, Egypt; g Department of Horticulture, Faculty of Agriculture, Ataturk University, 25240 Erzurum, Turkey; h Department of Food Technology and Nutrition, Makerere University, Kampala, Uganda; i Department of Biotechnology, College of Science, Taif University, P.O.Box 11099, Taif 21944, Saudi Arabia; j Marwadi University Research Centre, Department of Microbiology, Marwadi University, Rajkot, Gujarat 360003, India

**Keywords:** Sustainable meat production, Sustainable development goals, Adulteration detection, Meat quality assessment, Chemometric analysis, Spectral databases, Meat authentication, Non-destructive techniques

## Abstract

The growing global demand and price fluctuations in meat have raised concerns over safety, adulteration, and traceability. Conventional methods are time-consuming, labor-intensive, and reagent-dependent, limiting their use for rapid or on-site screening. This review provides a comprehensive overview of emerging non-invasive techniques—such as fluorescence, near-infrared, mid-infrared, and Raman spectroscopy—for assessing meat quality and detecting adulteration. The key novelty of this review is its integration of bibliometric analysis with a critical evaluation of advanced technologies aligned with the UN Sustainable Development Goals. The review also highlights the potential of hybrid systems that integrate spectroscopy with chemometrics and machine learning to provide accurate, real-time, and sustainable meat authentication solutions. It also highlights research gaps such as the need for multi-adulterant detection models, standardized validation protocols, and open-access spectral databases. By aligning innovation with regulatory and sustainability frameworks, this review advocates for robust, scalable solutions to build future-ready meat supply chains.

## Highlights

• Provides a critical comparison of conventional and emerging technologies for meat quality and safety evaluation.
• Showcases the power of AI-integrated spectroscopic method for rapid and precise meat adulteration detection.
• Detects key research gaps, highlighting the dire need for multi-adulterant detection frameworks in complex meat matrices.
• Hybrid analytical models outperform traditional ones in speed, sensitivity, and real-time applicability.
• Promotes tech-driven methods for meat authenticity, traceability, and alignment with UN Sustainable Development Goals.

## 1 Introduction

Meat stuffs serve as healthy food among the public and are widely consumed. Presently, the demand for healthy, natural and nutritious food including meat based products has increase (Alam et al., 2024; Saleem et al., 2024). In the past few decades, meat adulteration has become a significant problem in many countries and it has become a significant concern and critical task for meat processors to confirm meat quality (Adenuga & Montowska, 2023a; Liu et al., 2019). In the food industry and domestic retail markets, mechanically deboned meat has been widely used to produce different ground and restructured meat stuff (Candoğan, Altuntas, & İğci, 2021). However, the mincing process breaks muscle intactness and its mechanical properties making it quite challenging to detect the type of muscle, its origin and the presence of unidentified meat species (King et al., 2023). Another critical point in the literature on cultured meat is the lack of research recognizing the various priorities regarding the SDGs in economically diverse regions (less and more intensive agriculture/livestock countries) when introducing the in-vitro technology (Nobre, 2022). With a looming climate crisis, current climate action efforts remain far too slow and limited to effectively address the challenge. Extreme weather events now more frequent and severe are already affecting every region of the globe. As global temperatures continue to rise, these threats are expected to intensify, presenting serious and escalating risks (UN, 2023).

The integration of advanced, non-invasive technologies for meat quality assessment aligns with the broader objectives of the United Nations Sustainable Development Goals (SDGs), particularly in the context of climate action (SDG 13) and responsible consumption (SDG 12) (Sridhar et al., 2023). Advanced authentication methods help reduce food waste, enhance supply chain transparency and minimize the environmental impact of meat production a major source of greenhouse gas emissions. By promoting efficient, fraud-resistant systems, this work supports global efforts toward sustainable food systems and climate resilience. Fraudulent or substandard meat stuff not only pose a risk to consumer health (SDG 3) but also contribute to inefficient resource utilization and increased environmental burden due to unnecessary livestock production (Mills & Driscoll, 2022). By enabling early detection of adulteration and enhancing traceability through innovative approaches, such technologies support sustainable food systems, reduce waste and promote ethical practices in the meat supply chain. Moreover, they contribute to technological advancement and innovation (SDG 9), which are essential for building resilient and climate-conscious food industries (Ordoñez, Gonzales, & Corrales, 2024).

This intentional adulteration has both health and economic repercussions (Islam et al., 2022; Nolasco-Perez et al., 2019). The problem is not limited to the packing or processing industry but is apprehended at local retail shops and restaurants where this adulteration is easy to conceal. Adulterating meat of a specific species with meat from other species or plant-based ingredients affects consumers in diverse ways e.g.*,* food safety, religious observances, food allergy and economic loss. In this regard, adding soy protein to ground meat constitutes legal fraud if the added ingredients are not properly labeled on the packaging. In addition, incorporation of low-priced organ meat in minced meat and unlabeled mixing of various unidentified meats as horse, donkey, pork and likewise, is not uncommon but equally fraudulent (Elbarbary, Darwish, Fotouh, & Dandrawy, 2024). The presence of adulterated meat in the food supply chain endangers the beliefs and health of the people. Recently, several research groups have attempted to develop fast, accurate, comprehensive, reliable and real-time monitoring techniques to detect fraudulent and low-quality meat. In earlier studies it was reported that the three greenness tools, Sample Preparation Metric of Sustainability (SPMS), Blue Applicability Grade Index (BAGI) and the Analytical Greenness Metric for Sample Preparation (AGREEprep), were used to determine the health hazards, environmental sustainability values and applicability of this technique (González-Martín, Gutiérrez-Serpa, Pino, & Sajid, 2023; Manousi, Wojnowski, Płotka-Wasylka, & Samanidou, 2023; Wojnowski, Tobiszewski, Pena-Pereira, & Psillakis, 2022). In this regard, several spectroscopic, chromatographic and molecular approaches have demonstrated their capability to detect meat adulteration (Al-Murshedi et al., 2025). However, in developing countries, the potential of these advanced analytical methods for determining meat quality has not been fully explored. Despite the availability of rigorous regulations and principles regarding proper food labeling, misrepresentation of food products is commonly practiced to illicit monetary gains. Marketing of adulterated and unsafe meat is a societal concern due to continuously mounting prices of meat, augmented demand and escalating trade at the international level (Anagaw et al., 2024; Nolasco-Perez et al., 2019). Detection of adulterated, mislabeled, poor quality and spoilage meat is, however, still a big task for meat businesses which has pushed efforts to introduce rapid, innovative and trustworthy detection techniques to authorize safety, authenticity, and quality of meat based products (Ashaolu, Khalifa, Mesak, Lorenzo, & Farag, 2023; Cawthorn, Steinman, & Hoffman, 2013). Numerous studies have demonstrated that various advanced analytical techniques offer faster and more comprehensive results compared to traditional classical methods, thereby improving efficiency and accuracy in food analysis.

Among such techniques, polymerase chain reaction (PCR) is a well-known molecular method involving the chemical magnification of specific DNA sequences (Lee et al., 2024; W. Liu et al., 2019). This technique is beneficial in detecting different species in a mixed meat sample or detecting meat origin based on specific DNA sequences indigenous to different meat species (Abuzar et al., 2023; Adenuga & Montowska, 2023b). Relatedly, spectroscopic techniques, in combination with chemometric tools have demonstrated their potential to detect meat authenticity and invariably determine quality. For instance, several investigations have examined Fourier transform infrared (FTIR) spectroscopy to measure meat quality (Candoğan et al., 2021; Rohman et al., 2020). Similarly, fluorescence spectroscopy (FS) has demonstrated its ability to detect several types of plants and animal-based adulterants in meat. Thus, introducing these techniques in developing countries as rapid detection methods to combat the glitch of fraudulent meat marketing could increase both consumer confidence and public health (Cartin-Rojas, 2017; Maritano et al., 2024). The present review will address a comprehensive outline of the applications, over the last five years of different analytical (classical and novel) procedures along with multi-data analysis to determine the adulteration of meat stuff. This review concerns the prospects of novel detection techniques for meat quality and adulteration detection, and it focuses on the references published in the last five years with only a few older ones. The primary objectives of this review are as follows:

1. To provide an in-depth analysis of conventional methods for meat quality assessment and authentication, including chemical, biochemical, chromatographic, and DNA-based techniques. This section aims to highlight the strengths and limitations of these traditional approaches, which, although widely used, often require extensive sample preparation and are time-consuming.
2. To explore the development and application of non-invasive technologies such as fluorescence spectroscopy (FS), infrared spectroscopy (both mid- and near-infrared), and Raman spectroscopy in evaluating meat quality and detecting adulteration. This objective emphasizes how these advanced techniques offer rapid, reliable, and sensitive alternatives to conventional methods.
3. To examine the integration of non-invasive and conventional methods, assessing how the combination of both approaches can enhance accuracy, efficiency, and reliability in meat quality assessment. The review discusses the potential of hybrid models to overcome current limitations and address challenges in meat authentication and adulteration detection.
4. To identify current research gaps and propose future directions for improving meat quality and authenticity assessment by integrating novel technologies. This includes fostering innovation and advancing food safety and quality control practices.

## 2 Methodology

**Fig. 1:**
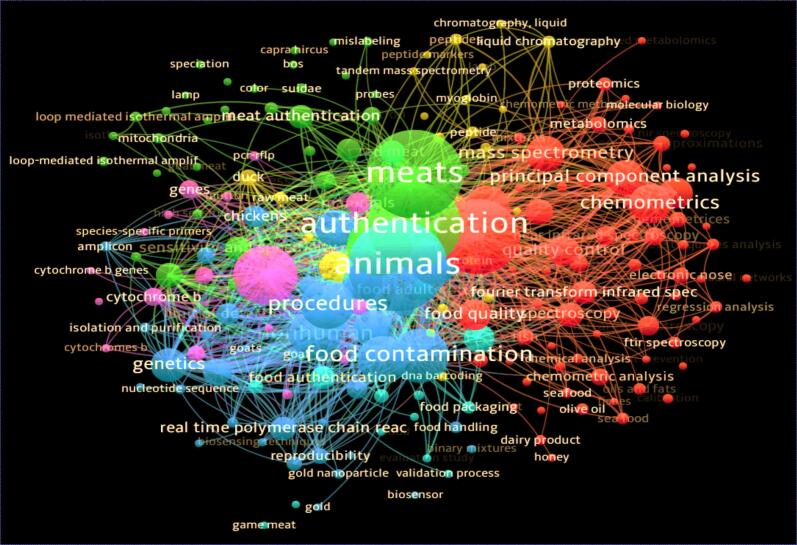
Bibliometric analysis of integration of non-invasive and conventional systems for quality assessment and authentication of meat and meat products. The data were acquired from Scopus database which was attached as a supplementary data. The figure was made using VOS-viewer software version 1.6.20.

To achieve these objectives, a comprehensive literature search was conducted using the Scopus database. The search strategy included keywords such as title-abs-key (“Meat” and “Adulteration” and “Authentication”) or title-abs-key (“Quality” and “Assessment”) or title-abs-key (“Chemometric” and “Analysis”) or title-abs-key (“Raman” and “Spectroscopy”) or title-abs-key (“Traditional” and “Methods”). Filters were applied to exclude irrelevant document types, non-English language publications, and pre-prints. This search yielded approximately 287 relevant articles (see Fig. 1) which illustrates a bibliometric keyword co-occurrence map derived from publications related to meat authentication, food contamination, and animal product traceability. The map was produced using VOSviewer based on titles, abstracts and keywords of peer-reviewed articles obtained from Scopus database. The extracted data were then systematically analyzed and narrated in the subsequent sections of this review.

### 2.1 Sensory evaluation

Sensory evaluation of meat stuff has a long history and is a fundamental tool to check the quality and contamination of meat (Table 1). In sensory evaluation of meat, meat flavor is a critical quality aspect, but because of meat matrix complexity and advancement, complete understanding of meat quality is still a challenge for food technologists (Xu & Yin, 2024). However, in the past few years, the detection and quantification of aroma compounds in meat has tremendously improved. Nearby, hundred aroma and flavoring compounds are present in meat, contributing to the flavor and complex attributes of meat palatability (Flores, 2023). Like sulfur-containing odor compounds, many of these compounds were altered due to cooking, storage, and non-meat ingredients. However, sensory evaluation of flavor is still functional in the food industry.

**Table 1:** A summary of sensorial and physicochemical methods used for determining the authenticity of meat-stuffs.

Meat products	Analysis purpose	Principle parameters	Main results	References
Fresh and frozen chicken meatballs.	To investigate the effect of chickpea and lentil flour as breading material on the properties of fresh and frozen chicken meatballs.	Sensory attribute, physicochemical, storage stability, pH, and color.	- Moisture retention and reduced oxidation stability compared to control (coated with corn flour). - pH, thiobarbituric acid, total volatile basic nitrogen, and free fatty acid values of the meatballs increased with prolonged storage. - Effect of storage on the sensory properties was not significant except for odor. L* value of color decreased while b* value increased during storage.	(Kilincceker & Yilmaz, 2019)
Meat-based products such as turkey breast, different sausages, and cooked ham.	To evaluate the effect of partially replacing NaCl by Soda-Lo® salt microspheres with different percentages (25, 30, and 50 %) on *W*/W bases.	Sensory or microbiological aspects.	No significant statistical difference was observed in control and replaced samples either in sensory or microbiologically for these different meat products.	(Raybaudi-Massilia et al., 2019)

Quail meatballs.	To investigate the use of extender (soy and rice flour) in the formation of quail meatballs.	Sensory characteristics of meatballs as well as compositional & quality analysis.	Rice flour extended meatballs also displayed decreasing trend in physicochemical and sensory properties with increasing concentration.	(Dhond et al., 2017)
Non-meat ingredients in meat.	Assessment of plant proteins in meat products.	Sensory and organoleptic properties.	Their sensory score showed that the increased addition of pea protein, at 3, 6, 9, and 12 % in meat, gave more off-flavor than the control. The tenderness of the meat also decreased at higher concentrations (12 % pea protein).	(Shoaib et al., 2018)
Meat sausages.	The impact of the inclusion of pork and green banana flour in meat sausages.	Sensorial, mechanical, physiochemical, and microbiological attributes.	Cooking loss and emulsion stability and maintained the products microbiological and sensory consistency improved by green banana flour and pork skin.	(dos Santos Alves et al., 2016)
Chicken meat from industrial and free-rang poultry systems.	To evaluate the quality parameters of chicken meat of both systems.	Classical methods including color, pH, proximate composition, and sensory evaluation were used for the comparison and evaluation of meat.	An opposite association between shear force, lightness & pH was observed. In comparison to the industrial broiler meat free-range broiler meat had higher yellow color and shear force and lower red color (a* and pH).	(da Silva et al., 2017)
Boneless and frozen beef.	To assess physicochemical parameters of boneless and frozen beef.	Fat, protein, water, pH, and ash.	Results showed that all chemical composition was satisfactory, except for the fat content.	(Ziani et al., 2018)
Beef burgers.	To investigate the physicochemical properties of beef burgers.	Texture, color, pH, volatile compound, chemical composition, and sensory evaluation.	Control and modified meat samples showed positive results.	(Szpicer et al., 2020)
Goat meat nuggets.	To check the impact of adding mushroom stem waste powder in meat nuggets.	Physicochemical quality, oxidative stability, sensory quality, texture, and color, including shelf life.	- The emulsion stability, dietary fiber, ash, emulsion stability, and phenolic content of nuggets compared to control were improved significantly by adding Mushroom stem waste. - Although in textural properties and expressible water no significant differences, moreover, color and sensory parameters did not negatively affect.	(Banerjee & Ray, 2019)
Low-fat chicken nuggets.	Development and physicochemical evaluation of low-fat chicken nuggets using extenders.	Physicochemical catalogs include protein (%), moisture (%), fat (%) and sensory attributes.	- Physicochemical showed a non-significant difference when compared with control. - Substantial difference was observed in sensory attributes, texture and overall acceptability.	(Singh et al., 2014)
Chicken meat.	Physicochemical and functional properties.	Fatty acid composition.	The quality of poultry meat has multivariate and intricate property.	(Milicevic et al., 2015)

For example, Raybaudi-Massilia et al. (2019) studied the sensorial properties of meat-based products such as turkey breast and different sausages. They cooked ham to evaluate the effect of partially replacing NaCl with Soda-Lo® salt microspheres. No significant statistical differences were observed in the sensory scores between control and replaced samples, implying the uselessness of this method for meat adulteration detection. Meanwhile, the sensorial results of chicken nuggets showed that nuggets were not affected by adding moringa flower extract (1–2 %) after storage at 4 °C for up to 20 d. Kilincceker and Yilmaz (2019) investigated the effect of chickpea and lentil flour as breading material on the properties of fresh and frozen chicken meatballs by sensory evaluation. Meatballs coated with lentil and chickpea flour were divided into two groups. The first was analyzed as a fresh fried product, and the second group was stored for four months at −13 °C. Stored samples were analyzed at 0, 15, 60, 90 and 120th d. The effect of storage on the sensory properties was not significant except for odor. In the same context, Dhond, Devangare, and Chappalwar (2017) investigated the use of rice and soy flour as an extender in the formation of quail meatballs. The sensorial score, flavor, appearance, juiciness, and texture decreased with an increased proportion of soy flour up to 2.5 %. Rice flour extended meatballs also displayed decreasing trend in sensorial properties with increasing its concentration.

However, the addition of non-meat ingredients alters meat's sensory and organoleptic properties. Assessment of plant proteins in meat stuff can also be detected by sensory perception of such adulterated products. In this framework, Shoaib, Sahar, Sameen, Saleem, and Tahir (2018) investigated the effect of pea and rice proteins added in meat to prepare nuggets. Their sensory score showed that increased pea protein, from 3, 6, 9 to12 % in meat, gave more off-flavor than control. The tenderness of the meat also decreased at higher concentrations of pea protein (12 %). The impact of incorporating green banana flour and pork skin on the mechanical, sensory attributes of bologna-type sausages has been studied by dos Santos Alves et al. (2016). He found that such additions can maintain the sensory consistency of the product.

### 2.2 Physicochemical analysis

The main traits of meat quality that can be evaluated are sensory, physiochemical, microbial, and nutritional properties and safety to meet this demand. Some recent studies on physiochemical analysis of meat for quality determination are described in Table 1.

Chicken meat from industrial poultry and free range systems were analyzed for its quality parameters in a study conducted by da Silva, de Arruda, and Gonçalves (2017) to evaluate the quality parameters of chicken meat from free-range and industrial poultry systems. Classical methods including color, pH, and proximate composition were used to compare and evaluate meat. Both industrial and free-range chicken meat showed PSE (pale, soft, and exudative) anomaly (L* ≥ 53). Higher yellow color (b* 11.56) and shear force (2.75 kg) were observed in free-range broiler meat in comparison to the industrial broiler meat while red color (a* 1.65) and pH (5.75) were lower. Total fat levels were higher in industrial broiler meat and protein levels were higher in free range broiler meat.

Later, Ziani, Khodja, and Khaled (2018) assessed the physiochemical parameters of boneless and frozen beef, which was imported from the Brazilian market. Different samples of meat taken from a different portion of the whole frozen chicken were analyzed for physicochemical parameters such as fat, protein, water, pH, and ash. Results showed that all chemical composition was satisfactory, except for the fat content, which depends on the consumer's taste. Similarly, Milicevic et al. (2015); Tomaševic et al. (2023) have studied the chicken meat properties under commercial handling in Serbia. During 2012, 48 samples of broiler thigh and breast muscles were collected from two farms for subsequent meat quality analyses. It was found that the treatment plan on the farm significantly modified the physicochemical properties, growth performance, and feed intake of chicken meat. Petersen et al. (2024) examined the fatty acid pattern of diet showed that the principal fatty acids were composed of monounsaturated fatty acid compared to saturated and polyunsaturated fatty acid, which indicates that the quality of poultry meat is a multivariate and complex in composition.

Recently, Szpicer, Onopiuk, Półtorak, and Wierzbicka (2020) explored the physicochemical properties of beef burgers to analyze the effect of oat β-glucan concentrates, which are used as a fat replacer in burgers. One of the main conclusions of their study was that the replacement or modification of β-glucan concentrates by fats could allow the exchange of traditional beef burgers with a healthier low-fat meat product with similar customer appeal. In a similar approach, Mphaphuli, Manhivi, Slabbert, Sultanbawa, and Sivakumar (2020) investigated the impact of adding mushroom stem waste (MSW) powder in goat meat nuggets by a series of analytical methods, namely physicochemical, textural, and color. The ash, phenolic contents, emulsion stability and dietary fiber of nuggets compared to control samples were improved significantly following the addition of MSW. Although no significant differences (*p* > 0.05) were observed for textural properties and expressible water among the formulations, but water holding capacity were improved by MSW powder. Again, color and sensory parameters were not negatively affected by the inclusion of MSW in treated meat nuggets. Singh, Chitra Sonkar, and Dorcus Messih (2014) assessed the influence of extenders (chickpea flour and soya chunk) on quality of low-fat chicken nuggets. The soya chunks and chickpea flour were added to minced chicken meat at various levels (5, 10, 15 and 20 %) and compared with the control. Moisture, fat, carbohydrates, protein, and ash contents showed non-significant difference (p > 0.05) with control. However, most physiochemical and biochemical methods are time-consuming, so applying them online during transportation, processing and storage is problematic (Awan, Butt, Haq, Ashfaq, & Suleria, 2017; Z. Zhang, Sun, Sang, Jia, & Ou, 2022). This is the root cause of the numerous efforts made recently to improve the excellent quality of meat measurement methods. Thus, it has found some solutions to all the shortcomings of the traditional model and can have a pleasing effect on the quality assessment of muscular foods by good toughness, speed, non-invasive or destructive assays.

### 2.3 Chromatographic analysis

Chromatographic analysis is a powerful separation technique used to reliably differentiate chemically similar compounds within complex food matrices (Zappi et al., 2023). Techniques such as high-performance liquid chromatography (HPLC) and gas chromatography (GC) are widely employed to detect a broad range of food constituents, including amino acids, proteins, carbohydrates, phenolic compounds, as well as volatile and semi-volatile substances (Albarri, Vardara, Al, & Önal, 2024). Recently, several chromatographic methods have been applied specifically to assess meat quality, as outlined below.

#### 2.3.1 High-performance liquid chromatography (HPLC)

**Fig. 2:**
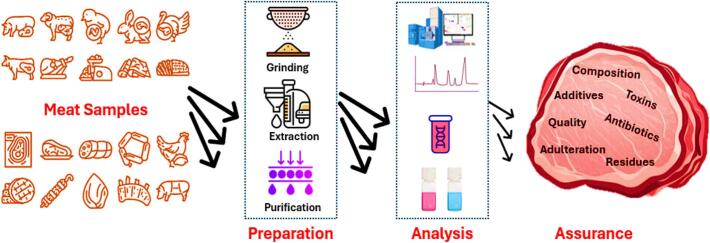
High-performance liquid chromatography for adulteration detection in meat-stuffs.

In analytical chemistry, HPLC is one of the most extensively used methods for determining the quality of food and food products. It is used in research and control tools routinely performed in industrial or laboratory settings because it can separate numerous chemical components of mixtures, characterize food products, or detect adulteration. Table 2 contained studies related to HPLC technique used for adulteration detection in meat (J. Zhang et al., 2024). Fig. 2 shows the pictorial representation of HPLC technique used for adulteration detection in meat stuffs.

**Table 2:** A summary of chromatographic methods (HPLC/GC–MS) used for determining authenticity and adulteration of meat-stuffs.

**Meat products**	**Analysis purpose**	**Analytical techniques**	**Main results**	**References**
Meat products.	Simultaneous detection of plant protein like (soy, pea, and lutein) from meat products.	HPLC –MS/ MS.	- High correlation of coefficient R^2^ > 0.9999. - Limit of detection for pea, soy, and lutein meat products was 5 mg/kg, 4 mg/kg, and 2 mg/kg, respectively.	(Hoffmann et al., 2017)
Meat products.	Detection of adulteration (oats, barley, rye, maize, rice, and wheat proteins) in meat products.	HPLC-MS/MS.	For every grain species, the LOD limits of detection of the technique were ≤ 5 or ≤ 10 mg grain protein/kg meat product.	(Jira & Münch, 2019)
Biogenic amines in meat.	Biogenic amines were determined in fish meat.	Column-switching high-performance liquid chromatography method coupled with fluorescence detector. HPLC-FID.	Seven different BAs (putrescine, tyramine, histamine, spermidine, 2-phenylethylamine, tryptamine, and cadaverine) were detected. High yielded, reproducible results were obtained.	(Ishimaru et al., 2019)

Mutton.	To identify adulterants in mutton.	*E*-nose/GC–MS.	GC–MS's findings confirmed that duck adulteration in mutton, with a minimum detection ratio of 10 % could be detected by E-nose.	(Wang et al., 2019)
Meat.	Identification of beef and pork.	GC–MS and UHPLC-MS.	Positive results in the identification.	(Trivedi et al., 2016)
Lard and beef tallow.	Detection in canola oil.	GC/MS.	5 % of lard and beef tallow spiked into canola oil were detected.	(Fang et al., 2013)

GCMS-HS: Gas chromatography-mass spectrometer with headspace analyzer, HPLC: High-performance liquid chromatography, UPLC: Ultra-performance liquid chromatography, EA-IRMS: Elemental Analyzer–Isotope Ratio Mass Spectrometry, HPLC-MS/MS: High-performance liquid chromatography - Mass Spectrometry, GC/MS: Gas chromatography-mass spectrometer, E-nose: Electronic-nose.

In the context of the detection of the adulteration of meat stuffs, Jira and Münch (2019) used HPLC-MS/MS for the detection of oats, barley, rye, maize, rice and wheat proteins in meat based products as these grain proteins commonly used in several types of meat samples (Sartagoda, Rhowell Jr, & Sreenivasulu, 2024). Grain-based protein produces emulsion-type sausages with blank values and in the 5–1000 mg/kg concentration range. For every grain species, the limits of detection of the technique were ≤ 5 or ≤ 10 mg grain protein/kg meat product and no false–negative or positive results were obtained. On the other side, Hoffmann, Münch, Schwägele, Neusüß, and Jira (2017) explored HPLC-MS/MS screening method's ability to simultaneously detect plant protein-like (soy, pea, and lutein) in meat stuffs. To conduct their research, the authors extracted plant protein followed by tryptic digestion and measurements of 3, 4 market peptides for each plant species by HPLC-MS/MS. Emulsion-type sausages were produced for matrix calibration with 0, 1, 6, 32, 160, 800 and 4000 mg/kg legume protein isolate. The mentioned legumes in sausages were detectable in the concentration of ≥6 mg/kg legume flour/isolate. Between the peak areas of the mass transition of the marker peptides and legumes protein contents in meat stuff, a high correlation coefficient R2 > 0.9999 was obtained. The detection limit for pea, soy, and lutein meat stuff was 5, 4, and 2 mg/kg, respectively. Another study was conducted by Ishimaru, Muto, Nakayama, Hatate, and Tanaka (2019) in which biogenic amines was determined in fish meat with the help of column-switching HPLC coupled with a fluorescence detector with isocratic of acetonitrile and water as mobile phases. This method detected putrescine, tyramine, histamine, spermidine, 2-phenylethylamine, tryptamine, and cadaverine biogenic amines with a high yielded and reproducible results.

#### 2.3.2 Gas chromatography (GC)

Gas chromatography (GC) is one of the most widely used separation techniques in food research for analyzing volatile compounds including pesticides, aromas and other trace chemicals. For decades, GC-based methods have been indispensable for chemical characterization, enabling the detection and identification of compounds at minute concentrations. It has been used to predict the fatty acids profile in meat and other substances such as oils, alcohols, pesticides, and drug analysis. For instance, GC was used for calculating the fatty acids content of meat samples (Yang, Zhang, Yang, & Xie, 2023).

Recent advancements have demonstrated GC–MS's effectiveness in meat authentication. The Wang et al. (2019) used GC–MS for identifying mutton meat. For this purpose, duck meat was chosen as an exemplary adulterant due to its similarity in flavor to mutton and its lower cost. Multilayer perceptron neural networks, fisher linear discriminant, and linear regression analysis were applied for quantitative and qualitative analysis on *E*-nose signals. GC–MS identified many fingerprints volatile organic chemicals. The findings of GC–MS established that E-nose (with a minimum finding ratio of 10 %) could detect duck adulteration in mutton with high accuracy and good detection efficiency with less detecting time. In the same context, Trivedi et al. (2016) assessed meat adulteration by developing new techniques using metabolomics to improve existing methods. Different marks of pork and beef mince bought from the meat outlet were mixed in several percentage ratios and evaluated using UHPLC-MS and GC–MS. By applying a series of chemometric tools, the authors differentiated between the two meat types. Through comprehensive chemometric analysis of various percentage mixtures obtained from commercial sources, the researchers established strong discrimination between meat types. These studies collectively emphasize the critical role of chromatographic techniques in food authentication, though additional investigation could further expand the reference database of chemical markers for different meat species and adulteration scenarios.

### 2.4 Molecular techniques for detection of meat quality and safety

**Fig. 3:**
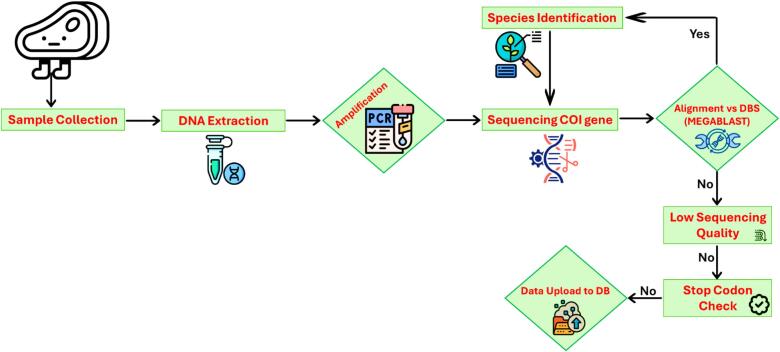
Flowchart illustrating the process of meat species identification using DNA barcoding.

For ensuring transparency in the meat industry, it is imperative to develop some reliable and more precise methods for accurate detection of meat quality in terms of authenticity in different products. In this regard, DNA-based techniques have been successfully employed to identify fraud meat. Several studies have been conducted to identify meat authenticity using PCR method as illustrated in Fig. 3. PCR is a classical molecular technique that has shown massive potential for detecting a small amount of DNA and can be used to measure the origin of meat species for deep-processed products (J. Li et al., 2022). Only some studies on use of PCR for adulteration detection in meat have been described in this section.

Kumari, Rank, Kumar, Joshi, and Lal (2015) postulated that real-time PCR techniques could detect meat authenticity. Their study used PCR for differentiating buffalo and cattle meat in mixed meat samples by targeting Cyt b gene variability and species-specific primers. Their results showed that real-time PCR could differentiate buffalo and cattle DNAs in mixed meat samples. Additionally, Cheng, Chou, Lee, and Sheu (2016) developed a PCR-based detection method for meat adulteration using RNA samples. The reaction targeted mRNA sequences of tropomodulin genes, troponin I (TnI), and mitochondrial ribosomal protein (MRP) specific for beef, chicken, goat, ostrich, and pork meat. The detection limits for cDNA were 0.01 ng and 20 ng in simplex and multiplex PCRs, respectively. Later, Amaral, Santos, Oliveira, and Mafra (2017) succeeded in detecting pork meat adulteration quantitatively in processed meat stuff with the help of real-time PCR. In their study, the development and optimization of the normalized assay were done based on the Ct method, allowing the detection and quantification of pork meat levels in raw and thermally processed meat as low as 0.0001 and 0.01 % (*w*/w). This method was validated using blind testing. The obtained results agreed with those of Ha et al. (2017), who succeeded in detecting the adulteration of pork meat in commercially processed chicken and beef meat samples. The authors developed a PCR method using species-specific primer for pork mitochondrial D-loop. Primers for specific species were taken for checking pure and adulterated meat samples. The results showed that the methods were able to detect as little as 1 % pork in heat-treated pork-beef-chicken mixtures, which helps prevent adulteration, which is unethical and has religious constraints. T. T. Li et al. (2019) used real-time PCR to develop a novel reference primer-based mitochondrial (12S rRNA) for calculable determination of pig adulteration in goat meat. The method exposed high sensitivity and specificity within 10–100 % for goat meat mixed with pork. Moreover, the authors effectively applied this method to identify commercial meat samples. In processed meat, the presence of chicken meat was identified with loop-mediated isothermal amplification (LAMP) assay developed by Sul, Kim, and Kim (2019), which has been successfully used for commercial meat monitoring. They designed five chicken primers (targeting mitochondrial 16S rRNA gene), including two outer two inner and one loop primers to identify chicken in processed meat; using LAMP techniques. Results stated that 0.1 % chicken meat in a raw meat sample and 1 % chicken meat in a heat- and pressure-treated meat sample could be identified with 30 min detection time.

As classical techniques (sensory, physiochemical, chromatography and molecular techniques) described above are expensive, laborious and time-consuming (Kaloo, Naqash, Majid, Makroo, & Dar, 2024). Thus, the demand for authentic and safe meat has increased interest in developing rapid analytical techniques like spectroscopic techniques described below.

## 3 Novel approaches for the determination of quality and authenticity/ adulteration of meat stuff

In the wake of increasing food fraud cases, particularly in meat and meat-based products, there is a pressing need for more reliable authentication techniques. Conventional analytical approaches, while effective, may lack the efficiency needed for high-throughput industrial applications. Therefore, new-generation methods are being explored to enhance accuracy, traceability, and consumer confidence.

### 3.1 Spectroscopic methods for the determination of quality and authenticity/adulteration of meat stuff (future need and trends)

Consumer requirements are increasing day by day for high-quality and safe food products. There is a need to improve or develop rapid techniques to eliminate the problems associated with classical techniques (Hassoun et al., 2023). In this regard, Spectroscopic techniques (fluorescence spectroscopy (FS), near infrared (NIR), mid infrared (MID) and Raman spectroscopy) are helpful for the determination of meat quality and authenticity because these are more rapid, simple, less expensive and non-destructive techniques. These spectroscopic techniques were used successfully to determine minced meat adulteration with textured soy protein, fat beef trimmings and horse meat (Aït-Kaddour et al., 2016; Edwards, Manley, Hoffman, & Williams, 2021). The combination of near-infra-red (NIR, MIR and Raman) with chemometric methods was used for monitoring and detecting meat adulteration. Recently, FS was used to detect meat adulteration (Du et al., 2023; Sul et al., 2019). The integration of these techniques offers a more comprehensive and reliable approach to determining meat quality and authenticity. For example, NIR can provide rapid screening of general composition, while Raman or MIR can offer detailed chemical insights into specific adulterants or meat species. FS can serve as an early indicator of spoilage, complementing the other methods by providing a fast, sensitive assessment of freshness. By combining these non-invasive techniques, it is possible to develop robust quality control systems that ensure the safety, authenticity, and quality of meat stuff, from production to retail (X. Wu, Liang, Wang, Wu, & Sun, 2022).

#### 3.1.1 Fluorescence spectroscopy (FS)

**Fig. 4:**
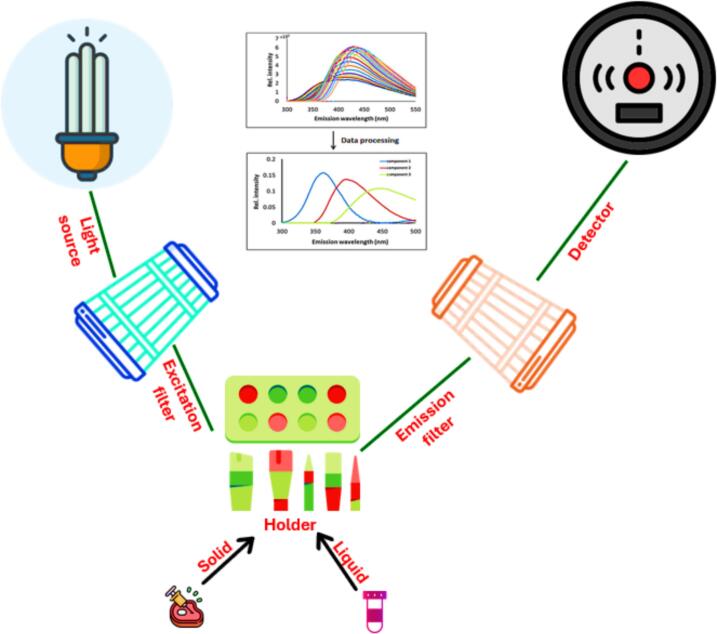
Schematic diagram of a fluorescence spectroscopy setup for meat analysis.

FS has emerged as an inexpensive, simple, rapid and non-destructive analytical technique for evaluating meat quality (Saleem, Sahar, Pasha, & Shahid, 2022; Wu et al., 2022). Recent advancements have enabled the integration of low-cost yet highly accurate fluorimeters with advanced software systems, allowing for real-time data acquisition, spectral magnification, and improved reproducibility. These developments have significantly enhanced the reliability and efficiency of fluorescence-based analysis. The fluorescence spectroscopy can detect subtle biochemical modifications (for example protein or lipid oxidation, microbial activity) at early stages, making it a potent tool for determining freshness and shelf life. Consequently, various fluorometric approaches have been successfully applied to assess the quality, safety and authenticity of meat and other food products (Asif, Tariq, Usman, & Sahar, 2025). FS is also a well-suited technique for direct food analysis as it does not require fractionation procedures that may change the product's nature. Fluorescence spectroscopy can also monitor changes occurring during food products' technological processes and storage conditions (Saleem et al., 2022). The obtained spectra of a product at specific excitation and emission wavelengths demonstrated different constitutional changes of a specific molecule as shown in Table 3 and Fig. 4. Innovation in the feature of spectrofluorometer with statistical tools increased its use in the field of food science. Meat contains many fluorescent compounds known as intrinsic or auto-fluorescence (Radotić, Stanković, Bartolić, & Natić, 2023). Some lipid oxidation products in meat are fluorescent compounds, pyridinoline in collagen, protoporphyrin IX, tryptophan residues in protein and vitamin A in meat fat (Saleem et al., 2022). The combined effect of FS and chemometrics has wide applications in authentication and classification of meat stuffs, as a function of their storage, cooking and processing circumstances and microbial spoilage. Quantitative assessments of some meat components (e.g., fat and fatty acids) were established using multivariate regression methods (Beriain, Ibañez, Beruete, Gómez, & Beruete, 2021; Li et al., 2019). Studies related to fluorescence spectroscopy for detecting meat quality are primarily focused on measuring the fluorescence of proteins (fluorophores; amino acids), collagen, vitamins, adipose tissues and nucleic acids (Fan & Su, 2022).

**Table 3:** Use of fluorescence spectroscopy technique for determination of authenticity and adulteration of meat-stuffs.

**Meat-stuffs**	**Analytic method**	**Analysis purpose**	**Wavelength**	**Statisticalmethods**	**Main results**	**References**
Beef muscles.	**Front-Face Fluorescence Spectroscopy**	Beef muscles Classification and characterization.	The excitation wavelength range of 250–550 nm using offsets (Δ*λmax*) of 20, 30, 40, 50, 60, 70, 80, 90, 100, 110, 120, 130, 140, 150, and 160 nm between excitation and emission wavelengths.	PLS-DA, POLAR.	96 % good classification with PLS-DA.	(Sahar & Dufour, 2015)
Bovine meat.	Synchronous front-face fluorescence spectroscopy SFFFS.	Monitoring of thermal changes in meat.	The excitation wavelength range of 250–550 nm using offsets (Δ*λ*_*max*_) of 20, 30, 40, 50, 60, 70, 80, 90, 100, 110, 120, 130, 140, 150, and 160 nm between excitation and emission wavelengths.	PCA & PARAFAC models.	98 % of the explained variance.	(Sahar et al., 2016)
Beef meat.	**Front-Face Fluorescence Spectroscopy**	Potential of fluorescence spectroscopy to predict the fatty acid composition. of beef.	For FFFS, the emission spectra were recorded at 305–400, 340–540, 360–570, 400–650, and 410–700 nm after excitation at 290, 322, 335, 350382 nm, respectively. For SFS excitation, fluorescence spectra were recorded between 250 and 550 nm, ix Δλ (i.e., 20, 40, 60, 80, 100, and 120 nm) were used.	PSLR.	Results suggested that the fluorescence spectroscopy is more suited to measure SFA (R2p ≥ 0.66; RPD ≥ 2.29) and MUFA (R2p ≥ 0.48; RPD ≥ 1.49) than PUF. (R2p ≤ 0.48; RPD ≤ 1.63).	(Aït-Kaddour et al., 2016)
Beef meat.	**Front-Face Fluorescence Spectroscopy** and Synchronous Fluorescence Spectroscopy	Performance of fluorescence spectroscopy for beef meat authentication.	For FFFS, emission spectra were recorded at 305–400, 340–540, 360–570, 400–650, and 410–700 nm after excitation at 290, 322, 335, 350382 nm, respectively. For SFS excitation, fluorescence spectra were recorded between 250 and 550 nm, ix Δλ (i.e., 20, 40, 60, 80, 100, and 120 nm) were used.	PLS-DA, PLS-SVM, PCA -SVM.	FFS, the PLS-SVM with the 382 nm excitation. wavelength gave the best Discrimination results (Recall, Precision, and Error of 94.34, 89.53, and 6.03 %, respectively). For SFS, when performing discrimination of the three muscles, the 120 nm offset gave the highest. Recall and Precision (from 57.66 to 94.99 %) and the lowest Error values (from 6.78 to 8.66 %).	(Aït-Kaddour et al., 2016)
Bovine meat.	3D fluorescence spectroscopy	For the assessment of bovine quality deterioration and freshness, including lipid oxidation color changes, and degradation of protein, and measures of reduced freshness.	(△λ = 75 nm, λex = 210–450 nm).	Cluster analysis (CA), pre-excitation-emission matrix (EEM) of adipose tissue along with the parallel factor analysis (PARAFAC) algorithm.	The calibration and verification accuracy of the obtained model is 95.56 and 93.33 %, respectively.	(Liu et al., 2019)
Fish.	**Front-Face Fluorescence Spectroscopy**	Identification and quantification of tuna species in canned tunas with sunflower medium	Em VS Ex 305–400: 290 340–540: 322 410–700: 382	FDA and PCA.	FDA was applied to the different intrinsic probes; the classification rates were not satisfactory. PCA showed correct classification amounting to 74.6 % on the calibration data sets.	(Boughattas et al., 2019)
Chicken and beef meat.	**Front-Face Fluorescence Spectroscopy**	Detection of chicken meat adulteration in beef meat.	Em VS Ex Em: 290, 322 and 340 nm Ex: 410 nm	PCA, PSLR, and CA.	Chicken meat adulteration can be detected in beef meat up to 10 % concentration level.	(Saleem et al., 2024)

Partial least square regression (PLSR), analysis of variance (ANOVA), Partial least square regression discriminates analysis (PLS-DA), Root means square error (RMSE), Principal component analysis (PCA), Standard error of the mean (SEM), Partial least square support vector machine (PLS-SVM), Principal component analysis support vector machine (PCA-SVM)**,** Factorial discriminant analysis (FDA)**,** Cluster analysis (CA), Excitation-emission matrix (EEM), Parallel factor analysis (PARAFAC) algorithm**.**

Sahar and Dufour (2015) conducted a comprehensive study to evaluate the applicability of fluorescence spectroscopy (FS) in distinguishing between different muscle types and assessing the rheological and physicochemical properties of meat. Key quality parameters including fat, protein, collagen, dry matter content, and texture were measured. Emission spectra were recorded across three ranges—305–400 nm, 340–540 nm, and 410–700 nm corresponding to excitation wavelengths of 290 nm, 322 nm, and 382 nm, respectively. The application of chemometric techniques such as Partial Least Squares Discriminant Analysis (PLS-DA) and Partial Least Squares Regression (PLSR) demonstrated that front-face fluorescence spectroscopy (FFFS), when integrated with multivariate statistical models, holds substantial potential for accurate identification and classification of meat muscle types. Later the same research group; Sahar et al., (2016) studied the possibility of SFS (synchronous front-face fluorescence spectroscopy) combined with chemometric tools to determine the effect of thermal treatments on cooked meat quality. Bovine samples were cooked at different temperatures at various times. Fluorescent spectra were obtained at excitation wavelengths varying between 250 and 550 nm and were studied using PARAFAC and PCA models. The results confirmed that the SFS gave data related to the arrangement of molecules in meat as a function of cooking temperature and time. The best PARAFAC model was achieved with three constituents, with 98 % of the explained variance and 55 % core consistency values and allowed to get information concerning the fluctuations in meat fluorescent compounds when cooked at 66, 90 and 237 °C and time (0–10 min.) (Jeneske, 2023; Li et al., 2021).

A non-destructive mobile system has been developed to monitor meat quality and possible applications in the entire fresh meat production chain (Sahar et al., 2015; Shi et al., 2021). Lamb and pork were stored at 5 °C for 20 d after death and measured with a FS. The fluorescence of NADH and different porphyrins may be associated with the growth of various bacteria to be used for pollution monitoring. According to the results, a mobile fluorescence system was set up for pork and lamb for two days in the afternoon and compared with the laboratory system. This mobile and non-invasive measuring system will increase the microbiological safety of fresh meat.

Aït-Kaddour et al. (2016) conducted a comparative study to evaluate the effectiveness of front-face fluorescence spectroscopy (FFFS) and synchronous fluorescence spectroscopy (SFS), both coupled with Partial Least Squares (PLS) modeling, in predicting the fatty acid profile and total fat content of longissimus thoracis (LT) muscles in beef. Samples were collected from 36 animals across three different cattle breeds. The findings indicated that fluorescence spectroscopy (FS) was more effective in predicting saturated fatty acids (SFAs) and monounsaturated fatty acids (MUFAs) compared to polyunsaturated fatty acids (PUFAs). Moreover, FFFS outperformed SFS in predictive accuracy, leading to the conclusion that FFFS is better suited for evaluating the lipid composition of beef meat.

Again the performance of SFS and FFFS coupled with PLS-DA, PCA-SVM and PLS (PLS-SVM) were investigated by Aït-Kaddour et al. (2016) to categorize three muscles of beef. Five excitation wavelengths were examined using FFFS, while for SFS six offsets Δλ_max_ were used. Comprehensively, results showed an excellent judgment among samples with precision and recall between 94.34 and 47.82 %.

H. Liu et al. (2019) examined the potential of 3D-FS technology in assessing bovine quality deterioration and freshness. The fluorescence properties of conjugated Schiff bases and amino acids can be also indicators of inner biochemical reactions due to beef spoilage, including lipid oxidation color changes, and degradation of protein, and measures of reduced freshness. Cluster analysis (CA) is used to classify beef quality by color (sensory index) and pH (chemical index). These results demonstrate the potential of FS inaccurately and non-destructively monitoring bovine quality deterioration.

Recently, Boughattas, Le Fur, and Karoui (2019) checked the authenticity of tuna fish using FFFS coupled with Factorial Discriminant Analysis (FDA) to examine the aromatic amino acids and nucleic acids (AAA + NA), tryptophan residues, nicotinamide adenine dinucleotide, riboflavin and vitamin A spectra, recorded on 232 canned tunas. Saleem et al. (2022) determined adulteration of chicken meat into minced beef mixtures using front face FS coupled with PCA, PLSR, and CA. These chemometric tools predict adulteration in minced beef meat up to 10 % chicken meat but were not good in distinguishing adulteration level from 1 to 5 %.

FS is overly sensitive to the intrinsic fluorescent compounds in meat, such as amino acids, proteins, and lipids. This technique can detect subtle changes in these components, making it useful for evaluating the freshness and spoilage of meat. For instance, the degradation of proteins or the oxidation of lipids due to microbial activity or improper storage leads to shifts in the fluorescence spectra. Moreover, FS can also detect the presence of additives, preservatives or adulterants that may alter the meat's natural composition. This makes it a promising method for ensuring authenticity by identifying deviations from the standard composition of meat stuff (Han et al., 2020). While fluorescence signals can vary substantially with type of meat, pH, fat content and additives, leading to inconsistent results and reduced reliability without careful standardization or calibration.

#### 3.1.2 Infrared spectroscopy

**Fig. 5:**
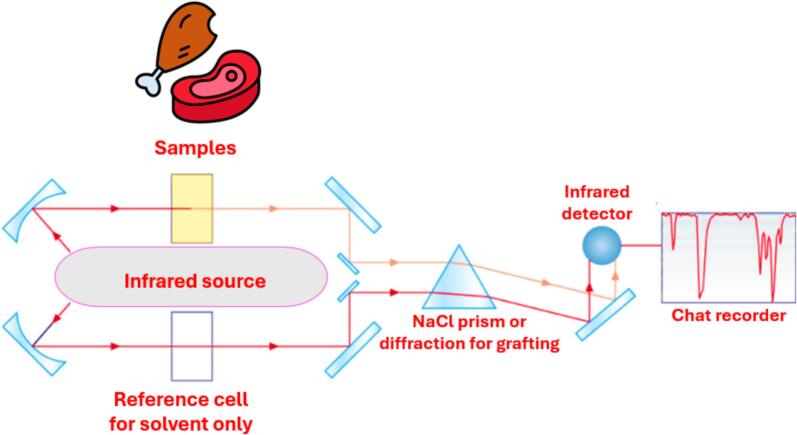
Infrared spectroscopy setup for meat analysis.

Absorption of incoming infrared radiation at a specific frequency by liquid, solid, liquid, or gaseous samples produces a spectral ‘fingerprint’ of the sample (Fig. 5). Infrared spectroscopy, also known as vibrational spectroscopy, coupled with chemometric analysis for qualitative data interpretation, has gained a reputation for checking meat genuineness and quality. The infrared radiation region has been divided into parts in the electromagnetic spectrum depending on the wavelength range: mid (MIR), near (NIR) and far-infrared. Several studies have been related to vibrational spectroscopy showing potential to define meat composition, principally fat content and fatty acid composition (Johnson, Walsh, Naiker, & Ameer, 2023; Tarcan & Küplülü, 2024).

##### 3.1.2.1 Near-infrared spectroscopy

The development of Near-Infrared Reflectance (NIR) spectroscopy has primarily been driven by the need for rapid analysis and adaptability to varying sample conditions (Table 4). It offers several advantages over conventional analytical techniques. As a non-destructive method, NIR requires minimal sample preparation, eliminates the use of chemicals or reagents, and generates no waste. Moreover, it delivers fast results with a high signal-to-noise ratio, typically around 10,000:1 (Büning-Pfaue, 2003; Fowler, Morris, & Hopkins, 2020). These attributes make NIR particularly suitable for industrial applications where speed, sustainability, and operational efficiency are prioritized.

**Table 4:** Use of NIR-infrared spectroscopy technique for determination of authenticity and adulteration of meat-stuffs.

**Meat-stuffs**	**Analysis method**	**Wavelength (nm)**	**Analysis purpose**	**Statistical methods**	**Main results**	**References**
Turkey meat and beef meat.	Fourier Transform- Near-infrared spectroscopy	800–2667.	Identification and quantification of turkey meat adulteration in fresh, frozen-thawed and cooked minced beef.	PLS	PLS regression models with R^2^ in prediction >0.884.	(Alamprese et al., 2016)
Beef meat fraud.	Fourier Transform- Near-infrared spectroscopy	800–2500.	Finding adulteration with horse meat.	PSL-R	62.5–100.0 % for whole meat, 75.0–100.0 % for minced beef with R^2^ = 0.85–0.94, SECV = 7.52–13.83 %, RPD = 2.2–5.7.	(Wiedemair et al., 2018)
Beef, turkey, and pork.	Visible and Near-Infrared Non-Invasive spectroscopy	400–1000 (near infrared) or 900–1700 (Vis-NIR).	Assessing different processed meats for adulterants plant and animal proteins as potential adulterants in minced beef and pork.	PSLR, LDA, PLSDA, MSC, SNV.	90 % of the rate was classified using pure while 100 % for adulterated ones. The correlation was found in 0.78 to 0.86 for optimal results.	(Rady & Adedeji, 2018)
Pig meat.	**Near-Infrared Spectroscopy** and classical methods.	400–2498.	Compared to the standard pH and color method of meat quality analysis, the potential of NIRS to predict the physical and chemical properties of LD muscle was evaluated.	PLS and RPD	R^2^ of the final pH was 0.67 and an RPD of 1.6. NIRS shows excellent potential for estimating the color parameter of porcine LD muscle.	(Furtado et al., 2018)
Pork and duck in ground meat.	Near-infrared spectroscopy	12,500–5400 cm^−1^.	To predict the adulteration and adulteration level of pork and duck in ground meat.	DA and PLS.	The DA model for wavelength selection gives the best results when the classification ratios of binary and triple systems are 100 and 91.5 %. The correlation coefficients Rp of the PLS model with the best purification level estimation of full wavelength are 95.80 and 95.69 %, orderly. RMSEP for binary and temporal samples is 7.27 and 9.27, respectively.	(Leng et al., 2020)
Pork meat in other meat samples.	Near-infrared spectroscopy	10,000 to 4000 cm^−1^.	To find the presence of pork meat in other meat samples.	PCA, PLS-DA, and PLSR.	The PLSR model was constructed with an R^2^ value of 0.9774 and an RMSECV value of 1.08 %. In addition, external validation was performed using the test set, which provided an outstanding prediction error with 1.84 % RMSEP.	(Mabood et al., 2020)
Ground beef.	**Visible/Near-Infrared** spectroscopy.	350–2500 nm.	To quickly determine the purity of ground beef. Beef adulteration.	SVM, RF, PLSR, and DCNN, PCA, SPA, LLE, and CARS.	RF with the significant wavelength spectrum selected by CARS provides the best performance for mixed pork, and the prediction coefficient (R^2^P) and RMSEP are 0.973 and 2.145. Use PLSR and CARS (R^2^P 0.960, RMSEP 2.758) to get the best beef estimate with beef.	(Weng et al., 2020)

Categorial data analysis (CDA), k-nearest neighbors algorithm (KNN), Modified partial least square (MPLS), linear discriminant analysis (LDA), PLS-DA Partial least squares discriminant analysis (PLS-DA), Root mean square error of prediction (RMSEP), Least square support vector machine LS-SVM, Mean square error (MSC), Standard normal variate (SNV), Soft independent modeling of class analogy (SIMCA), Support vector machine (SVM), Random Forest (RF), Partial least square regression (PLSR), Deep convolution neural network (DCNN), Principal component analysis (PCA), Sub window permutation analysis (SPA), Local linear nesting (LLE), Competitive adaptive re-weighted sampling (CARS), Near-infrared spectroscopy (NIRS), residual prediction deviation (RPD).

However, successful implementation of NIR spectroscopy depends on the robustness of calibration models and the availability of comprehensive reference datasets. While the technique provides speed and environmental benefits, it may exhibit lower sensitivity or specificity for certain analytes compared to traditional chemical assays. Therefore, stringent calibration and validation protocols are essential to ensure accuracy and reliability in practical applications.

These characteristics make it particularly valuable for industrial applications where speed and sustainability are prioritized. However, its implementation requires careful consideration of various factors. The technique's performance depends heavily on calibration models and reference datasets, while it excels in speed, it may trade off some sensitivity in comparison to traditional techniques. Moreover, proper instrument calibration and validation protocols are vital to ensure reliability.

Chiesa et al. (2016) has demonstrated NIR capability to determine the fatty acid profile of meat and Alamprese, Amigo, Casiraghi, and Engelsen (2016) studied the use of NIR, MIR and UV–Vis spectroscopy to identify the turkey meat adulteration in minced beef meat. Rady and Adedeji (2018) studied various meats like pork, beef, and chicken for authentication using NIR-spectroscopy. The potential of VIS-NIR spectroscopy was investigated by Prieto, Pawluczyk, Dugan, and Aalhus (2017) to discriminate regular beef cuts from dark beef cuts using commercial meat samples. López-Maestresalas et al. (2019) studied the use of NIR hyperspectral imaging to identify adulteration in minced lamb meat. Kuswandi, Cendekiawan, Kristiningrum, and Ahmad (2015) studied pork adulteration in beef meatballs by analyzing NIR spectral data using the chemometrics technique.

Adulteration of turkey meat samples was identified and quantified in minced beef by a group of researchers using FT-NIR spectroscopy and multivariate statistical method. Raw meat, frozen, thawed, and cooked meat samples were examined for the study. FT-NIR spectroscopy with chemometric strategies is a reliable tool for quantifying and identifying meat adulteration. In this study, different class modeling and multivariate regression were performed. PLSR models with R^2^ were evaluated with prediction more than 0.884 and RMSEP <10.8 %. To discriminate each type of sample in two classes, PLSDA was applied, showing the value of specificity and sensitivity in prediction more than 0.76 and 0.84, respectively (Fowler et al., 2020).

Wiedemair, De Biasio, Leitner, Balthasar, and Huck (2018) evaluated a total of 63 meat samples—comprising horse (*n* = 14), pork (*n* = 10), turkey (n = 10), chicken (n = 10), beef (*n* = 9), and mutton (n = 10)—using both Fourier-Transform Polarization Near-Infrared Spectroscopy (FT-NIR) and a portable micro-electromechanical system (MEMS) to assess their efficacy in detecting meat adulteration. After mincing, the samples were intentionally mixed to simulate adulteration scenarios, enabling comparative performance analysis of the two systems. In a related study, Rady et al. (2018) investigated the differentiation between plant- and animal-derived proteins introduced as adulterants in minced pork and beef using spectroscopic techniques. Through optimized wavelength selection, the study achieved 90 % classification accuracy for pure samples and 100 % accuracy for adulterated samples, with correlation coefficients ranging from 0.78 to 0.86, indicating strong model performance for authentication purposes.

Compared to the standard pH and color method of meat quality analysis, the potential of NIR to predict the physical and chemical properties of the longissimus dorsi (LD) muscle of pigs was evaluated by Furtado et al. (2018) with the pH results of the colorimeter and the pH meter. Spectral statistics of each sample (*n* = 77) as the average of 32 consecutive scans in the 400–2498 nm spectral range (2 nm range) for calibration and verification of the model were obtained. For each model (PLS), regression was used. R^2^ and the residual prediction deviation (RPD) of the predicted color parameters L *, a * and b * are 0.67 / 1.7, 0.86 / 2, and 0.76 / 1.9, respectively. R^2^ of the final pH was 0.67 and an RPD of 1.6. NIRS shows enormous potential for estimating a* color parameter of porcine LD muscle. More research on larger samples will help improve the quality of the model.

Leng et al. (2020) employed near-infrared (NIR) spectroscopy (12,500–5400 cm^−1^), a rapid and non-destructive technique, in conjunction with mathematical modeling to detect and quantify the adulteration of pork and duck in ground meat. By optimizing spectral wavelength selection and applying various spectral preprocessing techniques, the authors developed discriminant analysis (DA) and partial least squares (PLS) regression models. The DA model, without any preprocessing, yielded the highest classification accuracies achieving 100 % accuracy in binary systems and 91.5 % in ternary systems. The best-performing PLS model, based on full-wavelength data, produced prediction correlation coefficients (Rp) of 95.80 % and 95.69 % for binary and ternary adulteration levels, respectively. Additionally, the root mean square errors of prediction (RMSEP) were 7.27 and 9.27 for binary and ternary samples. These findings affirm the applicability of NIR spectroscopy not only for simple binary adulteration detection but also for more complex multi-component adulteration scenarios in minced meat sample.

In a similar study, Mabood et al. (2020) found pork meat in other meat samples using rapid analytical method NIR reflectance spectroscopy combined with multivariate analysis. Principal component analysis (PCA) showed similarities and differences between various meat samples, and partial least squares discriminator analysis (PLS-DA) showed big differences between pure pork and spiked meat samples. A partial least squares regression (PLSR) model was constructed to estimate the pork content in other meats with an R^2^ value of 0.9774 and an RMSECV value of 1.08 %. Weng et al. (2020) used visible/near-infrared (Vis / NIR) reflection spectroscopy and multivariate methods to quickly determine the purity of ground beef. First, the reflectance spectra of the different ground beef samples mixed were taken at 350–2500 nm. For adulteration type identification and level estimation, the support vector machine (SVM), random forest (RF), partial least square regression (PLSR) and deep convolution neural network (DCNN) were used. In addition, principal component analysis (PCA), sub-window permutation analysis (SPA), local linear nesting (LLE), and competitive adaptive re-weighted sampling (CARS) were used to eliminate redundant information. Results indicate that the combination of Vis / NIR reflectance spectroscopy and multivariate methods can quickly and accurately detect mixed minced meat.

Near-infrared (NIR) spectroscopy is extensively utilized for the compositional analysis of meat stuff, offering a rapid, non-destructive approach with minimal sample preparation. It effectively measures moisture, protein, fat, and carbohydrate content by detecting the absorption of light by molecular bonds such as O—H, N—H, and C—H, which are abundant in meat matrices. Beyond basic composition, NIR is also valuable for evaluating meat quality attributes including tenderness, water-holding capacity, and fat distribution. Notably, NIR has proven effective in detecting adulteration, enabling the differentiation of meat species and the identification of non-meat adulterants such as plant-based fillers or economically motivated substitutions. However, several critical factors influence the accuracy and reliability of NIR-based analysis. These include its reliance on comprehensive spectral reference databases for precise species identification, its relatively lower sensitivity in detecting trace levels of adulterants compared to more specific techniques like polymerase chain reaction (PCR), and the necessity for regular calibration to maintain instrument precision. Despite these considerations, the technique's rapid turnaround time and operational ease make it highly suitable for industrial applications, particularly in contexts involving routine quality assurance and regulatory compliance. NIR spectroscopy depends on detecting overtones and combination bands of molecular vibrations (mainly C—H, O—H, N—H), which are broad and non-specific. This makes it complicated to precisely identify or quantify individual compounds in complex meat matrices especially when several adulterants or subtle compositional differences are present.

##### 3.1.2.2 Mid-infrared spectroscopy

Mid-infrared (MIR) spectroscopy facilitates the analysis of food compounds by exploiting their absorptive properties, as functional groups within food molecules absorb photons at characteristic frequencies (see Table 5). The absorption features of food components are represented in MIR spectra according to their wavenumbers. These spectra consist of bands arising from fundamental atomic vibrations, primarily bending and stretching modes. In bending vibrations, atoms move away from their original bond axis, altering the angle between bonds. Conversely, in stretching vibrations, atoms remain aligned along the bond axis, but the interatomic distance changes as bonds elongate or contract. Alamprese et al. (2016) studied various samples like fresh, frozen, and cooked meat. The authors observed that MIR was a suitable and reliable tool for detecting turkey meat adulteration in minced beef in all sample forms, either in raw or frozen and cooked form.

**Table 5:** Use of Mid-infrared spectroscopy technique for determination of authenticity and adulteration of meat-stuffs.

**Meat-stuffs**	**Analytic methods**	**Analysis purpose**	**Wavelength (**cm^−1^**)**	**Statistical methods**	**Main results**	**References**
Fresh, frozen, and cooked meat.	Fourier Transform- Near-infrared spectroscopy and Mid-infrared spectroscopy.	To detect turkey meat adulteration in minced beef in all sample forms, either raw or frozen and cooked form.	12,500 to 3750 with a resolution of 8.	PLS.	R2 > 0.884	(Alamprese et al., 2016)
Meat products (pies, sausages, and burgers).	Fourier Transform- Mid-infrared spectroscopy.	Detection of pork in higher-value meat mixes.	900–1900.	PLS.	A useful tool for detecting adulteration.	(Abu-Ghosh et al., 2017)
The meat of different animal species.	Mid-infrared spectroscopy	To predict some chemical parameters such as protein and fat.	4000–400.	PLS.	Successful prediction of protein and fat with 34 modern techniques for food authentication levels with R^2^ of 0.7534 and 0.9173, orderly. A good result (R^2^ = 0.88) was achieved to approximate the lipid content where only one wavenumber (2925) was used.	(Rohman et al., 2020)
Fresh beef muscle meat *(M. semitendinosus).*	Mid-infrared spectroscopy	To detect fraud in beef.	4000–400	PLS-DA.	The multi-grade PLS-DA specifically detects each mixer provides satisfactory results for tripolyphosphate only (correct classification rate > 90 %). Two PLS-DA models distinguish between fake and non-fake meat and provide a high success rate (95 %). To verify the model's ability to detect untrained impurities, this latest model included 100 % of the new validation set (samples including 20 meat) maltodextrin.	(Nunes et al., 2020)

Partial Least Square (PLS), Soft independent modeling of class analogy (SIMCA), Partial least squares discriminatory analysis (PLS-DA).

MIR was used to predict some chemical parameters such as protein and fat. The researchers depicted that bands ∼2925, 1746, and 2854 cm^−1^, are connected to fat content, whereas those 3288, 1657, and 1542 cm^−1^ are related to proteins. The validation process endorsed a successful prediction of protein and fat with Modern Techniques for Food Authentication levels with R^2^ of 0.7534 and 0.9173, respectively. Abu-Ghoush et al. (2017) assessed the potential of FT-NIR to diagnose the presence of pork in higher-value meat mixes rapidly. For this rapid diagnostic method, the meat mixes were prepared by adding variable pork meat levels to beef in a concentration ranging from 5 to 90 %. The scanning of the sample through the MIR spectra was measured in the 4000–400 cm^−1^ region. The researchers applied PLSR with MIR and absorbance ratios at 1654/1745 cm^−1^, (1395 + 1450 cm^−1^) / 1175 and 1540 / 1745 cm^−1^ were associated with pork level suggesting the usefulness of MIR as a diagnostic, analytical signal for detection purposes. Nunes, Andrade, Almeida, and Sena (2020) introduce the development of a multivariate classification method based on MIR and partial least squares discriminatory analysis (PLS-DA) to detect fraud in beef. To increase the water holding capacity of meat, the frauds include the addition of sodium chloride, carrageenan and tripolyphosphate, which provide economic benefits. The multi-grade PLS-DA model that specifically detects each mixer provides satisfactory results for tripolyphosphate only (correct classification rate > 90 %). However, the two PLS-DA models that distinguish between fake and non-fake meat provide a high success rate (95 %).

MIR spectroscopy, like NIR, is based on the absorption of infrared light by molecular vibrations but operates in a different wavelength range that provides more detailed information about the molecular structure of meat components (Ozaki, 2021). MIR is highly effective in identifying specific functional groups in proteins, lipids, and carbohydrates, allowing for precise characterization of meat-based products. This technique can detect subtle chemical changes during meat processing, storage, and spoilage, providing insight into the overall quality. For authenticity and adulteration, MIR is particularly powerful in identifying meat species by comparing the unique molecular fingerprint of the meat with reference spectra. This helps prevent fraudulent substitution of high-value meats with lower-cost alternatives (Dashti et al., 2022). But the limitation is that meat has high water content and it absorbs water strongly in the mid-infrared region. This can hinder or distort key absorption bands of lipids, proteins and other analytes, making it complex to obtain distinct, quantitative data without sample preparation (for example drying or dilution).

#### 3.1.3 Raman spectroscopy

**Fig. 6:**
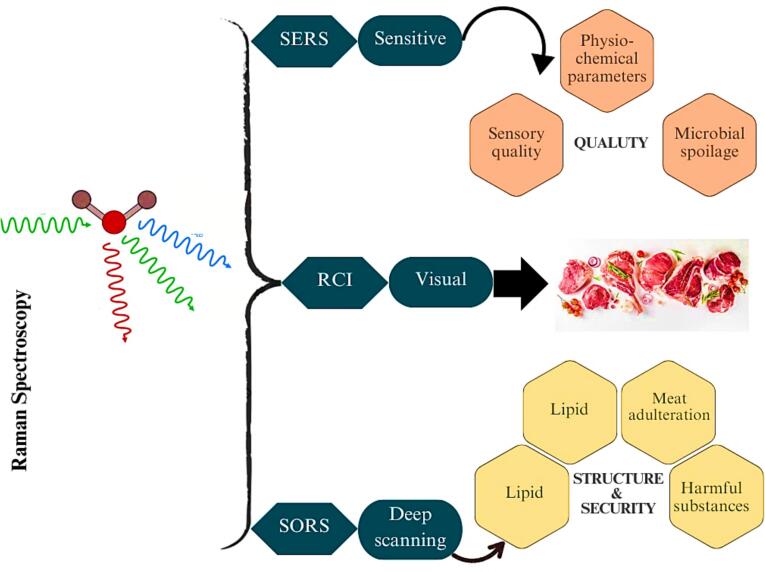
Applications of Raman spectroscopy in meat quality assessment.

One of the vibrational spectroscopic methods is Raman spectroscopy, which has been applied to determine the qualitative and quantitative analysis of meat stuffs (X. Wu et al., 2022) as illustrated in (Table 6 and Fig. 6). The NIR and MIR spectroscopies are based on the absorption process. Raman spectroscopy is based on the scattering method due to the diverse types of interaction of molecules with electromagnetic radiation. Inelastic light scattering is the basic theory of Raman spectroscopy. The absorption or transmission of light is the basis for infrared and NIR. The comparative reports X. Wu et al. (2022) demonstrated that Raman can reliably identify the meat spoilage and when combined with chemometric tools, distinguish between the species, like beef and horsemeat. The comprehensive review by Qu et al. (2022) highlights the rapid, in situ potential of Raman spectroscopy and its alternative (e.g., SERS, SORS) for assuring meat authenticity and safety. In contrast, the inelastic scattering of light is the foundation for Raman spectroscopy has high effectiveness for assessing food quality systems during processing, handling and storage (Abu-Ghoush et al., 2017; Sun, Tang, Zou, Meng, & Wu, 2022).

**Table 6:** Use of Raman spectroscopy technique for determination of authenticity and adulteration of meat-stuffs.

**Meat-stuffs**	**Analytic method**	**Analysis purpose**	**Wavelengths**	**Statistic methods**	**Main results**	**References**
Meat quality.	Raman spectroscopy.	Meat quality on pH bases.	500–1800 cm^−1^.	RMSECV.	RMSECV of 0.21 pH units which showed the virtual information.	(Niche et al., 2016)
Beef.	Raman spectroscopy (1300–2800 cm^−1^).	Sensory analysis of beef.	1300–2800 cm^−1^.	PSLR and RMSECV.	PLSR (R2CV: 0.63–0.89) and RMSECV: 0.38–6.88) for breed type.	(Zhao et al., 2018)
Meat quality.	Raman and NIR, fluorescence spectroscopic.	Post-mortem quality of meat.	400–2500 nm.	PSLR.	RCV2 (0.49–0.73) for all features examined with Raman spectroscopy. NIR and fluorescence spectroscopy displayed limited ability to analyze quality, with rcv2 (0.06–0.57) and (0.04 to 0.18).	(Andersen et al., 2018)
Beef.	Handheld Raman spectroscopic.	Measurement of sensory and quality parameters of (*M. longissimus lumborum*) beef loins.	500–1800 cm^−1^.		Predicted values of tenderness and juiciness (ρ) of 0.47 and 0.42, respectively.	(Fowler et al., 2020)
Beef quality.	Raman spectroscopy.	For prediction of intramuscular fat, Warner-Bratzler shear force, pH, drip, and cook-loss.	250–3381 cm^−1^.	PLS	The models exhibited superior calibration performance for pH (R^2^ ranging from 0.5 to 0.9 with low RMSEC values) and showed promising predictive capabilities for WBSF, IMF, drip loss and cook loss.	(Cama-Moncunill et al., 2020)

Single linear regression (SLR), partial least square regression (PSLR), Root means square error of cross-validation (RMSECV), principal component analysis (PCA), Warner-Bratzler shear force (WBSF), Intramuscular fat (IMF), NIR neat infrared spectroscopy.

In a different approach, Zhao et al. (2018) developed a fast-systematic procedure to envisage beef sensory aspects using Raman spectroscopy and to explore correlations between sensory traits using chemometric tools. Andersen, Wold, Gjerlaug-Enger, and Veiseth-Kent (2018) performed a study and recommended that spectroscopic procedures offer valued information about the post-mortem quality of meat. In their study, the authors used Raman spectroscopy, fluorescence spectroscopy, and NIR to evaluate drip loss, pH, and intramuscular fat in pork. From Raman spectroscopy, results were auspicious for PLSR, giving coefficients of determination from cross-validation (RCV2) (0.49–0.73) for all features examined. The NIR and fluorescence spectroscopy displayed limited ability to analyze quality, with R^2^CV ranging from (0.06–0.57) and (0.04 to 0.18), respectively. The authors concluded that Raman spectroscopy could be used to analyze the quality attributes of meat.

In one another current approach, Fowler et al. (2020) have worked using a handheld Raman spectrometer for the preliminary investigation to determine its potential to predict sensory properties as described by a group of untrained consumers. A 671 nm handheld Raman spectrometer was used to measure 45 pieces of beef fillet. The samples were then kept frozen until tested by an untrained sensory panel. Slices were also cut to determine the shear force value and other meat quality indicators. The model obtained shows that the Raman spectrometer can predict the juiciness and tenderness such that the predicted value and the observed value (ρ) are 0.42 and 0.47, respectively.

Cama-Moncunill et al. (2020) said that Speedy determination of beef quality stayed a challenge for meat handlers. To cope with this challenge, this study assessed the potential of Raman spectroscopy tracked by chemometrics for predicting intramuscular fat, Warner-Bratzler shear force, pH, drip, and cook-loss. Spectra recorded on frozen-thawed muscle and investigated by PLS regression models and validated using test sets randomly selected three times. According to the results, the loading values showed that the physicochemical deviation of the meat influenced the models. The results showed that Raman spectroscopy is a good technique for repetitive quality assessments of meat.

Raman spectroscopy is a complementary technique to MIR, as it also provides molecular-level information based on vibrational changes, but through the scattering of light rather than absorption (S. Zhang, Qi, Tan, Bi, & Olivo, 2023). Raman is overly sensitive to structural and compositional changes in proteins, fats, and other meat components, making it suitable for quality assessment by monitoring processes like fat oxidation, protein denaturation, and water content changes. One key advantage of Raman is its ability to analyze meat samples through packaging, making it a truly non-invasive technique. In terms of adulteration detection, Raman can identify chemical adulterants, such as synthetic additives or non-meat fillers, by detecting their unique spectral signatures. It can also be used to verify the species authenticity of meat stuff, ensuring that high-quality meat has not been replaced with lower-grade alternatives (M. Wu & He, 2024). Raman signals are often masked or overwhelmed by fluorescence emitted by certain substances in meat (for example heme pigments, fat or additives). This substantially reduces the signal-to-noise ratio, making it challenging to achieve clear and accurate spectral data particularly in fresh or processed meat samples. The comparative study of Qu et al. (2022), observed that portable Raman techniques such as SERS and SORS provide rapid, non-destructive insights into meat quality complementing NIR/MIR. Likewise, Boyacı et al. (2014) noted its precision in detecting 25 % horsemeat adulteration in beef using PCA-based chemometric analysis.

## 4 Conclusion

In summary, this review emphasize that the integration of classical analytical techniques with emerging non-invasive spectroscopic techniques represents a paradigm shift in meat quality assessment and authentication. Traditional methods continue to offer robust and validated benchmarks; however, their inherent limitations particularly in terms of time, cost and environmental impact reinforce the growing relevance of non-destructive, rapid and high-throughput technologies. The integrated application of these approaches supports the broader goals of sustainable development by promoting responsible production, ethical consumption, and climate resilience. Based on the authors' extensive understanding and critical evaluation of the existing literature, it is concluded that the future of meat quality assurance will depend heavily on the alignment of scientific advancement enabiling policy, infrastructure and industry practices to build a more transparent, efficient and sustainable food system.

Spectroscopic technologies such as fluorescence, near-infrared, mid-infrared and Raman spectroscopy combined with advanced chemometric and machine learning algorithms, offer promising solutions for rapid, sustainable and accurate meat analysis. The growing global research momentum, as revealed through bibliometric trends, highlights the critical importance of these advancements in combating food fraud, safeguarding public health, and ensuring regulatory compliance. However, the field would benefit from a more structured effort to validate hybrid detection models at scale, particularly under real-world industrial conditions. These models should be tested for reliability across diverse meat types, storage environments and levels of adulteration.

Additionally, the practical implementation of these technologies must consider their detection limits, particularly when dealing with trace levels of adulterants or spoilage indicators. Non-invasive methods such as NIR and Raman spectroscopy, while effective for rapid screening, may exhibit reduced sensitivity compared to molecular methods when applied at low adulteration levels. Moreover, their real-time applicability in industrial settings is often constrained by factors such as sample heterogeneity, processing speed and environmental interference. From a cost perspective, advanced spectroscopic instruments and required calibration protocols can pose substantial financial barriers for small and medium-scale enterprises, especially in developing regions. Regulatory challenges also remain a critical concern, as global standards for the validation, approval and harmonization of such technologies are still evolving. Addressing these issues through targeted research and policy alignment is essential for the successful large-scale adoption of these techniques in the meat industry.

Looking ahead, the development of hybrid detection models capable of real-time, on-site application will be pivotal in enhancing supply chain transparency and consumer trust. Equally essential is the establishment of standardized spectral databases and harmonized protocols to facilitate global adoption and interoperability. By embracing these innovations, the meat industry can not only elevate food safety and quality standards but also contribute meaningfully to the broader sustainable development agenda, thus promoting responsible production, ethical consumption and climate resilience. Ultimately, a strong alignment of science, policy and infrastructure will be essential in securing a safer, more transparent and sustainable food future.

## CRediT authorship contribution statement

**Asima Saleem:** Writing – review & editing, Writing – original draft, Validation, Resources, Investigation, Formal analysis. **Aysha Imtiaz:** Writing – review & editing, Supervision, Project administration, Investigation, Formal analysis, Conceptualization. **Sanabil Yaqoob:** Writing – review & editing, Writing – original draft, Visualization, Supervision, Methodology, Formal analysis, Conceptualization. **Muhammad Awais:** Writing – review & editing, Software, Resources, Investigation, Formal analysis. **Kanza Aziz Awan:** Writing – review & editing, Resources, Investigation, Formal analysis, Data curation. **Hiba Naveed:** Writing – review & editing, Software, Resources, Investigation, Formal analysis, Data curation, Conceptualization. **Ibrahim Khalifa:** Writing – review & editing, Writing – original draft, Supervision, Resources, Project administration, Conceptualization. **Sezai Ercisli:** Writing – review & editing, Visualization, Software, Investigation, Funding acquisition, Formal analysis, Data curation. **Robert Mugabi:** Writing – review & editing, Validation, Methodology, Investigation. **Saqer S. Alotaibi:** Writing – review & editing, Software, Methodology, Formal analysis. **Gulzar Ahmad Nayik:** Writing – review & editing, Supervision, Resources, Methodology, Investigation, Formal analysis. **Jian-Ya Qian:** Writing – review & editing, Visualization, Supervision, Software, Resources, Investigation. **Qing Shen:** Writing – review & editing, Visualization, Supervision, Resources, Investigation, Formal analysis, Data curation.

## Declaration of competing interest

The authors declare that they have no known competing financial interests or personal relationships that could have appeared to influence the work reported in this paper.

## Data Availability

No data was used for the research described in the article.
